# China’s Investments in Germany and the Impact of the COVID-19 Pandemic

**DOI:** 10.1007/s10272-021-0962-0

**Published:** 2021-04-06

**Authors:** Xinming Xia, Wan-Hsin Liu

**Affiliations:** 1 College of Urban and Environmental Sciences, University of Peking, Haidian District, 100871 Beijing, China; 2 Kiel Centre for Globalization (KCG), Kiel Institute for the World Economy (IfW), Kiellinie 66, 24105 Kiel, Germany

## Abstract

This paper analyses how China’s investments in Germany have developed over time and the potential impact of the COVID-19 pandemic in this regard, based on four different datasets, including our own survey in mid-2020. Our analysis shows that Germany is currently one of the most attractive investment destinations for Chinese investors. Chinese state-owned enterprises have played an important role as investors in Germany — particularly in large-scale projects. The COVID-19 pandemic has had some negative but rather temporary effects on Chinese investments in Germany. Germany is expected to stay attractive to Chinese investors who seek to gain access to advanced technologies and know-how in the future.

In early 2020, COVID-19 first caused a drastic lockdown of the Chinese economy. Subsequently, lockdown measures and further containment policies like business closures and mobility restrictions have been implemented worldwide to stop the spread of the coronavirus. The pandemic has led to a massive shock to the world economy (Ozili and Arun, 2020). Against this background, the United Nations Conference on Trade and Development (UNCTAD) expects that foreign direct investment (FDI) flows in 2020-2021 will drop by about 30% to 40% (UNCTAD, 2020a).

Decreasing FDI flows may make it more difficult for firms to acquire resources for their business and best satisfy the market needs. Such FDI decreases may further weaken the investment-based development impetus in many countries, impede the processes of idea exploration and research and development (R&D) and thus pose a threat to global prosperity.

* We would like to thank Frank Bickenbach and Holger Görg for their valuable comments on an earlier version of this paper and Michaela Rank for her research assistance. Xia would like to express gratitude to the Humboldt Foundation for its financial support for her one-year research stay at the Kiel Institute for the World Economy.

**Xinming Xia**, Peking University, China.

This paper provides empirical evidence of how China's investments in Germany have developed over time and of the investment impact of the COVID-19 pandemic. It also shows the relevance of China's policies in this regard. The analysis is based on four different data sources, including our small-scale survey in mid-2020 on the COVID-19 impact on FDI.

## Policy background: Outward FDI for China's development

Many Chinese firms investing abroad were motivated by their business interests (e.g. Cheung et al., 2012; Li et al., 2017; Wu, 2007). But their investment decisions and engagement have also been strongly guided and regulated by the Chinese government. China's economic development foci and strategies to overcome its economic challenges have been reflected in related Chinese policies that build the rules and regulations guiding Chinese investors' behaviour abroad (e.g. Child and Rodrigues, 2005; Morck et al., 2008).

China's 'Go Global' policy was announced in 1999 and marked a turning point in the Chinese government's attitude towards actively encouraging and supporting Chinese investments abroad (Rosen and Hanemann, 2009). Increasing investments abroad will help China to better deal with the appreciation pressure on the renminbi and more efficiently allocate the accumulated foreign exchange reserves.

With its accession to the World Trade Organization in 2001, China faced increasingly severe market competition. China thus increased its emphasis on the key role of science and technology and later also indigenous innovation and upgrading for enhancing Chinese firms' competitiveness and for sustaining China's economic growth in the long term. The Go Global policy was then formally integrated into China's national development strategy as reflected in the 10th Five-Year Plan (2001-2005) and reasserted in the 11th Five-Year Plan (2006-2010). Increasing Chinese investments abroad is intended to help Chinese firms gain better access to more advanced know-how and technologies, develop innovation capabilities and move up the global value chains.

With its strong economic development over decades, China became the second largest economic power worldwide in 2008 and the world export champion in 2010. China's economic growth has been, however, much weaker since then (Liu and Langhammer, 2016). New initiatives such as the Belt and Road Initiative (Felbermayr et al., 2019), Made in China 2025 (e.g., Garcia-Hererro et al., 2020; Zenglein and Holzmann, 2019) and the National Innovation-driven Development Strategy (Central Committee of China's Communist Party and State Council of China, 2016) were carried out to explore new development stimuli to deal with the emerging growth challenges. In the new wave of innovation promotion as a national strategy, private firms have received more attention than ever before. These initiatives co-shaped China's policies for guiding and encouraging outward FDI, which gained a much clearer regional and industrial/technological focus of FDI destinations or targets than before to help achieve the national long-term development goals. A key FDI guiding and regulating document announced by the National Development and Reform Commission (NDRC et al., 2017) clearly shows that Chinese outward investments that are more advantageous for supporting China's developing strategies such as its Belt and Road Initiative as well as structural change towards high-tech and advanced manufacturing are encouraged, while the investments in, e.g. real estate, sports and entertainment sectors are rather restricted.[1]

Considering the developing trends in China's Go Global Policy, Europe and, particularly Germany, now clearly belong to the attractive destinations for Chinese outward investments. On the one hand, the abovementioned Belt and Road Initiative aims to improve China's connectivity with Europe. On the other hand, the advanced countries in Europe such as Germany are potential sources of valuable know-how and technologies that are crucial for China to move up the global value chains and to achieve innovation-driven growth and technological leadership in the long term. On 30 December 2020, after years of negotion, the EU and China finally concluded in principle the EU-China Comprehensive Agreement on Investment. Such an agreement will further enhance the attractiveness of Europe (and Germany) for Chinese investments in the future, although it is still highly uncertain whether the agreement can be adopted and ratified and how long it will take until it can really be enforced.

## Research data

To learn more about how China's investments in Germany have developed over time and how they have been affected by the coronavirus pandemic, four different datasets with their unique strengths are analysed in this paper. First, the data from the Annual Statistical Bulletin of China's Outward Foreign Direct Investment (MOFCOM et al., 2007-2019) helps to provide an overview of the development of Chinese investments abroad in general and those in Germany in particular. Second, the data from the Investment Project Database provided by MOFCOM gives information about the distribution of all approved Chinese investment projects in Germany by ownership and by sector. Third, the China Global Investment Tracker (CGIT) provided by the American Enterprise Institute and the Heritage Foundation (2020) is used to deepen and update the investment distribution analysis by focusing on Chinese large-scale investment transactions in Germany. Last but not least, our own survey "Potential Impacts of the COVID-19 Pandemic on the Chinese Investment in Germany" also provides insight on the topic.

Different from many previous studies (e.g. Knoerich, 2010) for which investor data were analysed, our survey addressed consulting firms and organisations in Germany that have been intensively engaged in providing FDI consulting services to Chinese investors in mid-2020.[2] Since

1 In 2014/2015, several bureaus in charge of regulating Chinese investments abroad such as MOFCOM (2014) and SAFE (2015) simplified the regulation measures and approval procedures for Chinese investment projects. The Chinese government considered, however, a part of the strongly expanding Chinese outward FDI as inefficient and thus turned to strengthen its guiding and regulating role in 2017 (Huang and Tang, 2017). NDRC et al. (2017) clearly indicate the directions of Chinese investments abroad that are encouraged, restricted or prohibited. Chinese investments abroad in support of the Belt and Road Initiative and upgrading Chinese exports, in high-tech areas and advanced manufacturing, in energy and resources areas, in agricultural cooperation and in the finance sector are explicitly encouraged. In addition, state-owned enterprises (SOEs) are also required to better manage and supervise their enterprises abroad to enhance their investment quality and efficiency (MOF, 2017).

2 The list of invited firms and organisations is based on sector-related information collected from the Chinese Chamber of Commerce in Germany, the German-Chinese Business Association, the Investment Platform China/Germany and the German-Chinese High-Tech Alliance. We also consulted an expert in the consulting sector in Germany to further extend our list to include smaller key players less visible on the public platforms.

Figure 1
**Chinese investment flow to Germany**

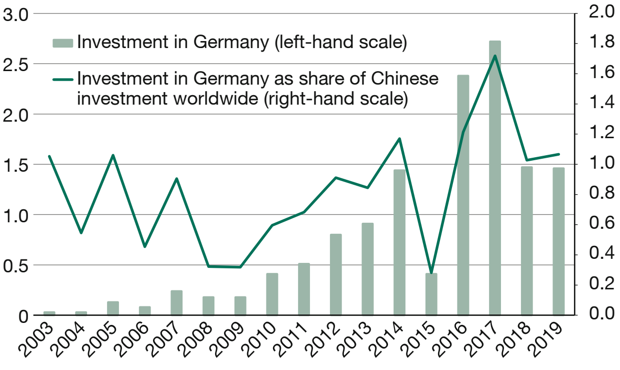

Source: MOFCOM et al. (2007-2019).

Figure 2
**Number of Chinese investment projects in Germany by investing firm ownership**

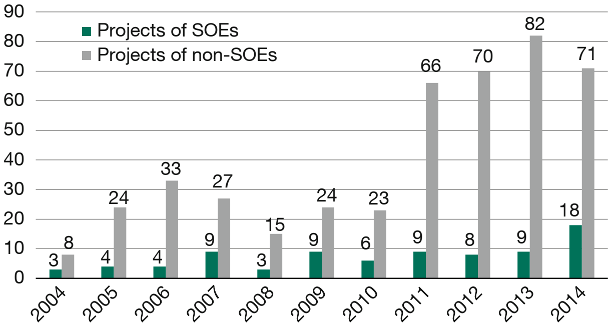

Note: SOE stands for state-owned enterprises.

Source: China's Investment Project Database provided by MOFCOM.

they consult not only investors who are already active in Germany, but also potential investors and those who left or are leaving the German market, our survey can help to provide a broader overview of whether and how Chinese investors may change their engagement in Germany in response to the COVID-19 pandemic. In total, 71 consulting firms and organisations in Germany were invited to join our survey, and 18 questionnaires (25%) were completed and returned. The small-scale survey might not be representative, but it can still provide some up-to-date information for business and policy orientation. The majority of these 18 firms and organisations are small in size in terms of the number of employees who were involved in providing consulting services related to Chinese investments in Germany at the end of 2019 (56%: 1-9 persons; 39%: 10-25 persons).

## Chinese investments in Germany: Key developments

Since the beginning of the new century, even against the backdrop of the global financial crisis and a significant slowdown in long-term global economic growth, Chinese investors continuously increased their FDI. The outward FDI flow substantially increased from about $0.9 billion in 2000 to $117 billion in 2019. In 2015, China's outward FDI flow even exceeded its inward FDI for the first time. With its outward FDI stock of $2,100 billion in 2019 (about 75 times its outward FDI stock in 2000, $28 billion), China is the third largest FDI source country in the world (UNCTAD, 2020b).

### Germany is one of the most attractive FDI destination countries for Chinese investments

China's outward FDI stock in Germany reached more than $14 billion at the end of 2019. Although this was only about 0.7% of China's total FDI stock in the world, Germany was ranked tenth among all destination countries of the Chinese investments (in stock) in 2019 (MOFCOM et al., 2019).[3] Chinese investors substantially increased their FDI flow to Germany after the global financial crisis (Figure 1). From 2009 to 2017, the Chinese outward FDI flow to Germany increased continuously at a much stronger rate than Chinese outward FDI in general, with the year 2015 being an exception. As a result, the share of Chinese investments in Germany in its world total clearly increased from 0.32% in 2009 to 1.72% in 2017. As mentioned above, the Chinese government strengthened its guiding role and regulatory power for Chinese overseas investment projects in 2017. This led to a substantial reduction in the Chinese investment flow to Germany in 2018 that remained stable in 2019 against the backdrop of further decreasing Chinese investments abroad in general.[4]

### Chinese SOEs played an important role as investors in Germany – particularly in large-scale projects

Figure 2 shows that there were 89 approved Chinese investment projects in Germany in 2014, compared to 11 projects ten years earlier.[5] A continuous increase in FDI projects can be observed after the global financial cri-

3 The top three destination countries/regions are well-known stop-over destinations and/or offshore financial centres: Hong Kong (58%), the Cayman Islands (12.6%) and the British Virgin Islands (6.5%).

4 The relatively strong decrease in China's investments in Germany compared to the world that led to a reduction in the corresponding share in 2018 can also be attributed to the increasingly strict supervision over non-EU FDI in Germany. In 2018, the German government for the first time prohibited an acquisition of a German firm by a Chinese company on national security grounds and prevented another acquisition attempt by a Chinese electricity giant.

5 The time period is determined by China's Investment Project Database provided by MOFCOM. The database is not further updated in a comparable way after 2014.

Figure 3
**Chinese large-scale investment projects in Germany by investing firm ownership**

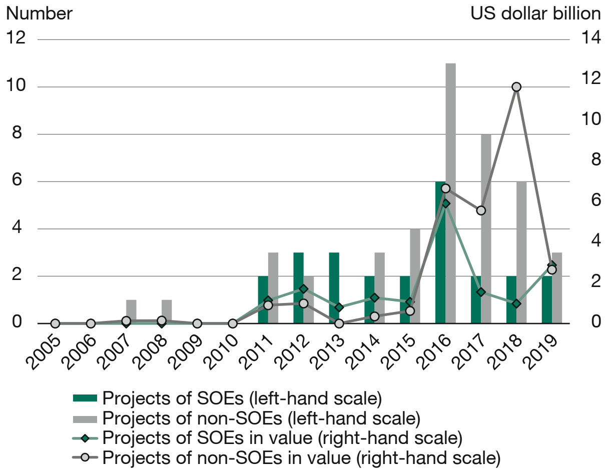

Notes: Projects with at least $100 million in investment. SOE stands for state-owned enterprises.

Source: American Enterprise Institute and the Heritage Foundation (2020).

sis. While Chinese state-owned enterprises (SOEs) accounted for three of the 11 approved investment projects in 2004, they only carried out 18 of 89 approved projects in 2014.[6]

SOEs play a much more important role as investors of large-scale (at least $100 million) investment transactions in Germany (Figure 3). From 2011 to 2014, they accounted for a significantly higher share of large-scale investment transactions in Germany than in case of the approved FDI projects in Germany in general (Figure 2). In the recent past, when the Chinese government again strengthened its guiding and regulating role in Chinese investments abroad, the number of Chinese SOEs' large-scale projects in Germany strongly decreased from 2016 to 2017 but stayed constant after that. The number of large-scale projects of non-SOEs decreased more strongly in the same period. The average size of the SOEs' large-scale investment transactions was larger than that of their non-SOE counterparts in all years since 2011, except for 2018.[7]

## Large-scale Chinese investments in Germany increasingly focused on transport and high-tech sectors

From 2005 to 2014, the large-scale investment transactions by Chinese investors in Germany were mainly targeted in the real estate sector (seven of the 20 projects in total), followed by the transport sector (five projects).[8] While the transport sector clearly gained in importance as a target industry after 2014, the attractiveness of the real estate sector substantially decreased. From 2005 to 2019, 20 of 25 large-scale transport investment projects (80%) in Germany were carried out in the last five years, compared to 30% in the real estate sector (three out of ten projects) in the same period. The investment projects in the transport sector in the last five years accounted for more than 92% of the total investment value in this sector from 2005 to 2019.[9]

The technology sector was ranked third in terms of project number and value for the large-scale investment transactions for the whole period considered. Similar to the transport sector, this was a result of a strong increase in attractiveness of the technology sector among large-scale investment projects by Chinese investors in Germany after 2014. Five of the six (83%) investment projects and 89% of the $5.9 billion investment amount in this sector from 2005 to 2019 were realised in the last five years.

The increasing importance of the transport sector and the technology sector as target industries of Chinese large-scale investments in the recent past is not surprising. As described above, in this period the Chinese government particularly encouraged outward FDI projects in support of China's new initiatives such as its Belt and Road Initiative and its need for structural change towards high-tech and advanced manufacturing.

Both SOEs and non-SOEs had large-scale transport investment projects in Germany and non-SOEs played a more dominant role in this regard (Figure 4). Non-SOEs were responsible for 13 of the 20 (65%) large-scale transport investment projects from 2015 to 2019 (75% measured in value), when the transport sector gained attractiveness as a target industry. Although the transport sector was also an important target industry for SOEs' investment in Germany (50% of their large-scale investment projects in number from 2015 to 2019), the average size of the SOEs' transport investment projects was smaller than

6 SOEs refer to enterprises with capital injection from the central and/or local governments (National Bureau of Statistics of China, 2003).
7 CGIT is able to identify the final recipients of Chinese investments, even if they have been made via third countries (Scissors, 2019). Comparing Figure 3 with the first two figures suggests that a great amount of Chinese FDI in Germany have been carried out indirectly, i.e. via third countries and this is particularly the case for Chinese SOEs as investors.
8 If all Chinese investment projects that were approved by the Chinese government are considered, i.e. not focusing on large-scale investment projects only, two services sectors played a highly dominant role: wholesale and retailing as well as leasing and commercial services.
9 Three of the 25 large-scale investment projects in the transport sector from 2005 to 2019 were related to the aviation field, while the others targeted at the automotive fields covering different parts of the supply chain of the automotive industry, such as automotive parts and components, engines and automatic control, and whole car production.

Figure 4

**Value of Chinese large-scale investment projects in Germany by target industry and investing firm ownership**

in billions of US dollars

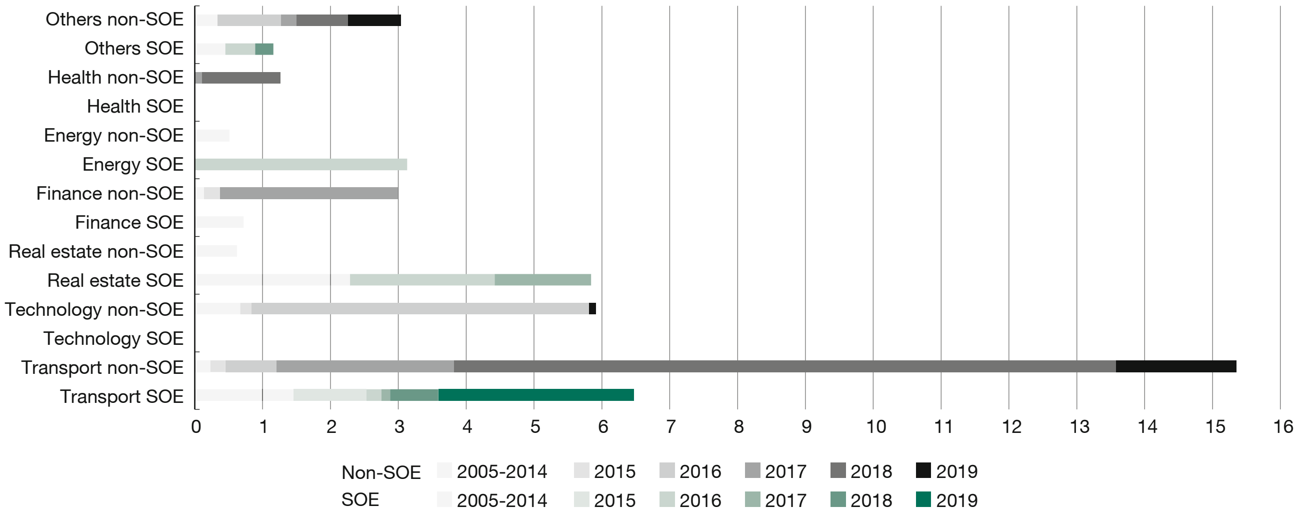

Notes: Projects with at least $100 million in investment. SOE stands for state-owned enterprises.

Source: American Enterprise Institute and the Heritage Foundation (2020).

that of their non-SOE counterparts, accounting for only about 40% of SOEs' total large-scale investment value in this period.

The role of non-SOEs was even more prominent in large-scale investment projects in the technology sector in Germany. They were responsible for all six such projects for the whole period from 2005 to 2019, including five that were carried out from 2015 to 2019. In contrast, SOEs were clearly dominant in Chinese large-scale real estate investments in Germany. They carried out eight of ten such projects for the whole period and accounted for more than 90% of the corresponding investment value, completely dominating in this area from 2015 to 2019 (three of three projects). SOEs' dominance in real estate investment projects in Germany and the stricter restrictions upon such projects imposed by the Chinese government provide some additional explanation to the strong decrease in SOEs' large-scale investment projects in Germany from 2016 to 2017.

## The COVID-19 pandemic has a negative but temporary impact on Chinese investments in Germany

Eighteen of 71 invited interviewees joined our small-scale survey. Twelve of them indicated that they received fewer general enquiries from Chinese investors regarding investment in Germany for the first half year of 2020 compared to the same period in 2019. They indicated that the COVID-19 pandemic had some but not a strong impact on such decline in general investment enquiries.

Such decline seems to be mainly driven by a decreasing number of Chinese investors' enquiries about new investment projects in Germany. As shown in Figure 5, the majority of the 18 interviewed firms and organisations observed a decline in enquiries about new investment projects in Germany by Chinese investors in the first half of 2020 compared to the same period in 2019. On the contrary, for the other investment/divestment purposes, a high share of firms and organisations interviewed did not observe significant change over the same research period. Despite this, still more than one-third of the firms and organisations indicated that the enquiries by Chinese investors for expanding their business operations in Germany – in scale (36%) or in scope (44%) – decreased in the same period. However, only a (much) smaller share received more enquiries by Chinese investors about scale reduction (13%), scope reduction (23%) and market exit (31%).[10] The findings suggest that at the time of the COVID-19 pandemic, Chinese investors turned out to be more cautious particularly regarding new investment projects in Germany. They may also hold off their investment expansion projects. But many of them may not immediately choose divestment.

10 The increase in enquiries for market exit as observed by about 31% of consulting firms and organisations interviewed is likely to be related to the fact that the Chinese investment projects in Germany (in terms of project number) have been traditionally concentrated in wholesale and retailing as well as leasing and commercial services. These sectors have been facing immediate market demand challenges due to strict containment measures amid the pandemic.

Figure 5

**Perceived development in Chinese investors' enquiries by investment purpose, first half of 2020**

Share of firms and organisations

Notes: Compared to the same period in 2019. Numbers in parentheses are the total number of firms and organisations that provided corresponding services to their clients.

Source: Authors' own survey.

Figure 6

**Perceived specific development in Chinese investors' enquiries by investment topic, first half of 2020**

Share of firms and organisations

Notes: Compared to the same period in 2019. Numbers in parentheses are the total number of firms and organisations that provided corresponding services to their clients.

Source: Authors' own survey.

Figure 7

**Expectations about development in Chinese investors' enquiries by investment purposes, first half of 2021**

Share of firms and organisations

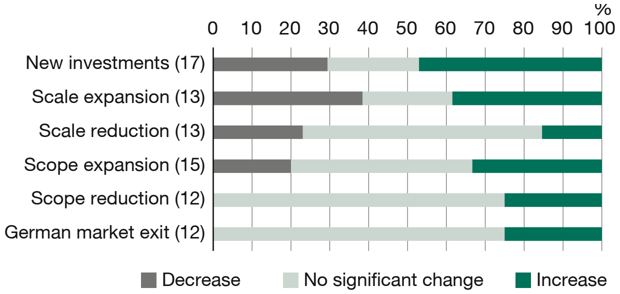

Notes: Compared to the same period in 2020. Numbers in parentheses are the total number of firms and organisations that provided corresponding services to their clients.

Source: Authors' own survey.

In line with the findings of a reduction in enquiries about new investments as observed by many consulting firms and organisations, the business registration services were less in demand in the first half of 2020. Almost half of the consulting firms and organisations that provided such services observed such a decline in development (Figure 6). Many of them (40%) also received fewer enquiries from Chinese investors for searching investment locations.

Consistent with the finding that Chinese investors may not immediately decide for divestment, but for problem-solving, many consulting firms and organisations observed increasing enquiries from the Chinese investors for consulting services for employment and labour issues (67%), financing and liquidity problems (50%) as well as supply chain disruption between the EU and China (60%). For the other consulting services, a clearly dominant share of the firms and organisations surveyed did not perceive significant change in enquiries from Chinese investors over the research period.

Firms and organisations interviewed tend to be optimistic as to their future business perspective (Figure 7). Focusing on consulting firms and organisations that also answered the survey question as to the current change in investment enquiries (Figure 5), almost half of them expect an increasing number of enquiries about new investments in Germany in the near future. Almost 40% (34%) of firms and organisations expect more enquiries from Chinese investors to expand their investment projects in scale (scope) in Germany. The shares of firms and organisations expecting more enquires for divestment in the future are smaller and they are hardly different from those in the early crisis period.

## Conclusions

The analysis presented in this paper shows that Chinese investments in Germany increased strongly after the global financial crisis, when China's policies shifted towards intensively encouraging quality- and innovation-based economic growth with outward FDI as an important instrument for Chinese firms to better access know-how and advanced technologies from abroad. Chinese SOEs were found to play a more important role in large-scale projects than in small-sized projects in Germany. The transport sector and the technology sector have become particularly more attractive to Chinese investors in large-scale projects since 2015 – the year of the release of China's strategy Made in China 2025. Our survey results suggest that the COVID-19 pandemic had some but not a strong negative impact on Germany's attractiveness for

Chinese investments and that such a negative impact is expected to be short-lived. Our findings are in line with the expectation that Germany, with its clear strength in high-tech, smart manufacturing and innovation activities, has become an attractive target for Chinese investments with their developments being strongly guided and regulated by Chinese policies.

The closing communiqué of the Central Committee of the Chinese Communist Party suggests that promoting innovation will continue to be one of China's top policy priorities. China will, inter alia, continue to work on modernising its industries and supply chains as well as on strengthening its manufacturing capability to pursue quality-based and innovation-driven growth in the future (Central Committee of China's Communist Party, 2020). Against this background, it is to be expected that Chinese policies will further encourage Chinese investments abroad that can help China to achieve its future innovation-driven development goals. Thus, Germany will probably continue to be attractive to Chinese investments in the future. This can be advantageous for sustaining Germany's future economic growth. A key question is, however, whether and how Germany should deal with Chinese investment attempts where the Chinese government plays a strongly guiding role. On the one hand, it is essential for Germany to develop adequate FDI policies to ensure its openness towards (Chinese) FDI so that Germany and its industries can further benefit from the foreign capital inflows and probably enjoy a fairer Chinese market access in return. The EU-China investment agreement may help here. On the other hand, while staying open to Chinese investments, addressing national security concerns without curbing Chinese investments in an unnecessary way is a critical challenge to Germany's FDI policies. Germany will also need to ensure fair competition, especially in mergers and acquisitions. Last but not least, how German firms and industries facing intensified competition from China can increase their competitiveness and whether and how the German government should support them in doing so will remain an important question for Germany's industrial policy.
